# Intravenous Lidocaine Modulates the Perioperative Hepatic Inflammatory Response: Implications for Personalized Medicine in Thoracic Surgery

**DOI:** 10.3390/jpm15120620

**Published:** 2025-12-11

**Authors:** Ana Isabel Galve, Ignacio Garutti, Elena Vara, Guillermo González, Gabriel Cusati, Lisa Rancan, Luis Huerta, Javier Casanova, Carlos Simón

**Affiliations:** 1 Anesthesiology Department, Hospital General Universitario Gregorio Marañón, 28007 Madrid, Spain; ignacio.garutti@salud.madrid.org (I.G.); gabrielcusati@gmail.com (G.C.); javier.casanova@salud-juntaex.es (J.C.); 2 Department of Biochemistry and Molecular Biology III, Faculty of Medicine, Complutense University of Madrid, 28007 Madrid, Spain; evaraami@ucm.es (E.V.); guillermo.gonzalez@salud.madrid.org (G.G.); lisaranc@ucm.es (L.R.); 3 Thoracic Surgery Department, Hospital General Universitario Gregorio Marañón, 28007 Madrid, Spain; luhuema@ucm.es (L.H.); carlos.simon@salud.madrid.org (C.S.)

**Keywords:** systemic response, liver inflammation, one lung ventilation, thoracic surgery, lidocaine

## Abstract

**Purpose**: Lung resection surgery (LRS) induces a strong local and systemic inflammatory response that may extend to peripheral organs, including the liver. This study aimed to evaluate the potential effect of intravenous lidocaine on hepatic inflammatory and apoptotic responses during lung resection surgery with one-lung ventilation (OLV) in an experimental porcine model. **Methods**: Eighteen mini pigs were randomly assigned to three groups: lidocaine (LIDO), control (CON), and sham (SHAM). Animals underwent left caudal lobectomy. The LIDO group received a continuous intravenous infusion of lidocaine (1.5 mg/kg/h) during surgery. The CON group received the same volume of saline, and the SHAM group underwent thoracotomy without lobectomy or OLV. Different samples were collected at baseline, during surgery, and 24 h postoperatively to assess inflammatory cytokines and apoptosis-related proteins. Liver biopsy was taken 24 h after de surgery. **Results**: One-lung ventilation and lung resection surgery increased the expression of proinflammatory markers in the liver biopsy and enhanced apoptotic protein expression and iNOS production. Lidocaine administration attenuated these effects, showing lower levels of inflammatory mediators, a better balance between iNOS and eNOS, and reduced apoptotic activity compared with controls. **Conclusions**: Our findings suggest that intravenous lidocaine may serve as a personalized perioperative strategy to attenuate systemic inflammatory and apoptotic responses, contributing to improved hepatic protection during thoracic surgery.

## 1. Introduction

Lung resection surgery (LRS) induces a local inflammatory response that can become systemic when proinflammatory mediators reach the bloodstream and eventually peripheral organs, including the liver [1]. This effect is enhanced when one lung ventilation (OLV) is required [2].

The OLV is necessary to perform certain surgical procedures in thoracic surgery, it contributes to biotrauma and also to ischemia/reperfusion (IR), especially in the ventilated lung, while surgical manipulation mainly affects the operated lung [3].

Proinflammatory substances cause damage related to oxidative stress in lungs and increase the levels of inflammatory markers in plasma, such as IL-1, IL-6 and TNFα [4,5].

Clinical manifestations of acute hepatic damage are rare in LRS; however, the liver plays a crucial role in systemic inflammation and is especially sensitive to IR [6,7]. Exposure of hepatocytes and Kupffer cells to circulating cytokines and oxidative stress can impair detoxification and amplify systemic inflammation. Consequently, postoperative multi-organ dysfunction, including acute respiratory distress syndrome (ARDS), may result from this inflammatory cascade [8].

Controlling perioperative inflammation has become a key goal in precision perioperative medicine. Identifying modulatory drugs such as lidocaine may help develop patient-specific anesthetic regimens aimed at reducing organ damage and improving outcomes. Numerous therapeutic strategies have been studied [9]. Lidocaine, in addition to its local anesthetic and antiarrhythmic properties, has anti-inflammatory effects by modulating the inflammatory response, inhibiting neutrophil activation, cytokine release, and oxidative stress [10,11]. This quality has been observed at different levels of the inflammatory cascade, which has serious consequences in the liver, where different types of lesions are produced, such as intracellular and interstitial edema and necrosis of hepatocytes [11].

The anti-inflammatory effect of lidocaine during surgical interventions is well known [12,13,14,15]. Previous studies from our group showed that lidocaine attenuates the lung inflammatory response during LRS [16]. However, its effects on hepatic inflammation and apoptosis, and the molecular mechanisms involved, remain incompletely understood [17].

Therefore, we hypothesized that lidocaine could modulate and prevent hepatic inflammation during LRS.

The main goal of our study was to investigate the impact of intravenous lidocaine administration during LRS on hepatic inflammatory, oxidative, and apoptotic responses during LRS with OLV, using an experimental porcine model.

## 2. Materials and Methods

This was an experimental, randomized, and blinded study conducted with the approval of both the Research Committee (CI) and the Animal Ethics Committee (CEEA) of the General University Hospital Gregorio Marañón (HGUGM). All procedures complied with current legal regulations, particularly Law 32/2007 of 7 November on the care of animals used for farming, transport, experimentation, and sacrifice, as well as Royal Decree 53/2013 of 1 February, which sets forth the fundamental standards for the protection of animals used in research and other scientific or educational purposes. The experimental protocol adhered strictly to Spanish and European Union legislation concerning the welfare and ethical treatment of laboratory animals.

### 2.1. Experimental Model

Eighteen mini pigs with an average weight between 30 and 40 kg were included in the study and underwent left thoracotomy for caudal lobectomy performed under OLV. Animals were randomly allocated to groups using a computer-generated randomization list. Laboratory investigators performing molecular analyses were blinded to group allocation.

The sample size (n = 6 per group) was based on previous studies from our laboratory showing consistent inter-group differences in inflammatory markers in similar porcine models. Power analysis indicated that this sample size provides adequate power to detect a significant difference in the analyzed markers.

We obtained three groups: lidocaine (LIDO), control (CONTROL), and sham (SHAM). In the LIDO group, animals received an initial intravenous bolus of lidocaine 1.5 mg/kg followed by a continuous infusion of 1.5 mg/kg/h maintained until the end of the surgery. In the CONTROL group, the animals received an equivalent volume of 0.9% saline solution. In the SHAM group, no OLV or lobectomy was performed, only thoracotomy and two lung ventilation.

Plasma lidocaine levels were not measured in this study; however, the dosing regimen follows clinically validated perioperative protocols and produces plasma concentrations within the therapeutic range reported in the literature. The same samples (blood, lung, and liver samples) were taken for analysis in all groups at baseline, 120 min after OLV, 60 min after TLV, and 24 h after surgery.

### 2.2. Anesthesia Protocol

A standardized anesthetic protocol was used to treat all animals in the same way.

An 18 h fasting period was performed, and only water was allowed freely up to two hours before the procedure.

Premedication consisted of an intramuscular ketamine 10 mg/kg (Ketolar^®^, Parke Davis, Pfizer, Dublin, Ireland). After premedication, the animals were positioned supine on the operating table, and continuous monitoring of the electrocardiographic and pulsioximetry was initiated. Anesthetic induction was achieved using fentanyl 3 µg/kg (Fentanest, Kern Pharmaceuticals, Houston, TX, USA), propofol 1% 4 mg/kg (Diprivan, AstraZeneca, Macclesfield, Cheshire, UK), and atracurium 0.6 mg/kg (Tracrium, Glaxo Smith Kline, Brentford, UK). Antibiotic prophylaxis was administered with benzathine penicillin (600,000 IU IM).

Maintenance was performed with continuous perfusion of propofol 2% (Diprivan^®^, AstraZeneca, Macclesfield, Cheshire, UK) and boluses of fentanyl and atracurium as required. Maintenance fluid therapy was administered with crystalloids, Ringer’s lactate (Hartmann Braun, Barcelona, Spain) 5–6 mL/kg/h.

Following induction, orotracheal intubation was performed with a No. 6.5 cuff endotracheal tube positioned 2–3 cm above the carina to perform two-lung ventilation (TLV). The correct position of the tube is checked by a fiberoptic bronchoscope (3.7 mm flexible Karl-Storz fiberoptic bronchoscope, with working channel).

The IOT tube is connected to the respirator and volume-controlled mechanical ventilation was performed with protective lung ventilation parameters: FiO_2_ 60%, tidal volume (TV) of 8 mL/kg, 6 mL/kg during OLV, peak pressure < 30 cmH_2_O, PEEP of 5 cm H_2_O, inspiration-expiration ratio of 1:2 and respiratory rate 12–15 breath per minute to maintain normocapnia.

Respiratory parameters, capnography, peak pressure, mean pressure, and lung compliance were monitored. When OLV was required (in LIDO and CONTROL groups), the proximal part of the IOT tube was connected with a second Murphy tube number 8.5–9 and advanced to the main right bronchus under fiberoptic bronchoscope vision.

When the procedure was completed, the animal was awakened and extubated. During the postoperative period, ketorolac 30 mg (Droal^®^, Vita S.A., La Paz, Bolivia) and dexketoprofen for pain were prescribed, and free access to water was allowed. At 24 h postoperatively, we performed another anesthetic induction in the same conditions in order to collect samples; after that, the animal was euthanized.

### 2.3. Surgical Protocol

The animals were positioned in right lateral decubitus, after which a left thoracotomy was carried out. Cannulation of the right femoral artery and vein was then performed. Through the arterial access, a venous line and a PiCCO thermodilution catheter (model PV2014L16, small adult femoral type, 4 Fr diameter, 160 mm length) were inserted for hemodynamic monitoring.

One-lung ventilation (OLV) was initiated in both the LIDO and CONTROL groups, and a caudal lobectomy was performed. After 120 min of one-lung ventilation (OLV120), TLV was restored for 60 min (TLV60). After performing all the determinations, and once TLV was restarted, the expansion of the left cranial lobe was confirmed, and the integrity of the bronchial suture was examined to ensure the absence of air leakage. Subsequently, the thoracotomy was closed in anatomical layers, leaving an intrapleural drainage tube connected to a one-way flow device (Heimlich valve).

After waking up the animal and 24 h after the intervention, it was transferred back to the operating room, and general anesthesia was performed again, following the same anesthetic protocol described above. Next, samples were collected: gasometric and hemodynamic study, blood extraction for biochemical studies, liver biopsy, and biopsies from the left cranial lobe and the mediastinal lobe.

To facilitate taking liver biopsies, prior to placing the animal in lateral decubitus, a subxiphoid median laparotomy of about 5 or 6 cm was performed.

Once we had the necessary samples, the porcine model was euthanized.

**Figure 1:**
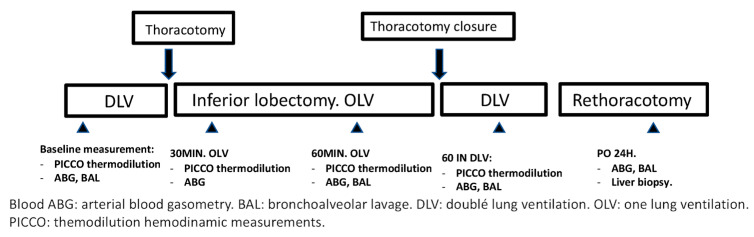
Experimental protocol design.

Experimental protocol design (Figure 1):

Hemodynamic data were collected through the PiCCO monitor, and transpulmonary thermodilution was performed periodically. Arterial blood gases were performed at different times during the procedure: baseline, 30 min of one-lung ventilation, 120 min of one-lung ventilation, 60 min after restarting two-lung ventilation, and 24 h after lobectomy.

BAL was performed at baseline, at 120 min of VUN, at 60 min after restarting TLV, and at 24 h after lobectomy. The recovered liquid was centrifuged and frozen at −20 °C until used.

### 2.4. Liver Samples

During the experiment, one liver sample was taken from each experimental animal to carry out the pertinent biochemical studies. The liver biopsy was taken 24 h after the lobectomy.

Each sample was placed in a cryotube, frozen in liquid nitrogen, and stored in a freezer (−80 °C) until its subsequent analysis.

## 3. Determination of Biochemical Markers

### 3.1. Proinflammatory Mediators: Cytokines

Concentrations of TNF-α, IL-1, IL-10, and MCP-1 in liver biopsy samples were determined using porcine-specific ELISA kits (Cusabio Biotech Co., Wuhan, China, and MyBiosource, San Diego, CA, USA), following the manufacturers’ protocols. In summary, both standards and samples were dispensed into antibody-precoated wells, allowing the target proteins present in the samples to bind to the immobilized antibodies. After washing away any unbound material, a biotin-labeled antibody specific to each analyte was added. Subsequently, avidin conjugated with horseradish peroxidase (HRP) was applied. Following further washes to eliminate residual reagents, a substrate solution was introduced into each well. The reaction was stopped after 10 min, and the absorbance was read at 450 nm. The intra-assay coefficient of variation was maintained below 8%, and the inter-assay variability remained under 10%.

### 3.2. Nitric Oxide

Nitric oxide production was quantified as the total concentration of nitrite plus nitrate (NO_2_^−^ + NO_3_^−^), using the Griess reaction. This assay determines nitrite levels after enzymatic conversion of nitrate (NO_3_^−^) to nitrite (NO_2_^−^). Briefly, plasma samples were incubated with *Escherichia coli* nitrate reductase and NADPH^+^ for 30 min at 37 °C. Following incubation, 300 µL of Griess reagent—composed of 0.5% naphthylenediamine dihydrochloride, 5% sulfonamide, and 25% phosphoric acid (H_3_PO_4_) (Sigma-Aldrich, Waltham, MI, USA)—was added to each sample. The reaction mixture was maintained at 22 °C for 20 min, after which absorbance was read at 546 nm using sodium nitrite (NaNO_2_) as the standard curve reference.

The assay showed a linear response between 1 and 150 mM (r = 0.994, *p* < 0.001, n = 5), with a detection limit of 2 µM. Experimental reproducibility was verified in three independent assays, each performed in triplicate. The mean intra-assay variation was below 5%.

### 3.3. Messenger RNA Expression (mcp-1, il-1, tnf-α, il-10, nf-κb, inos)

It was performed using the reverse transcriptase polymerase chain reaction (RT-PCR) technique.

Total RNA was extracted from porcine liver tissue following the protocol originally described by Chomczynski [18], employing the TRI Reagent kit (Molecular Research Center, Inc., Cincinnati, OH, USA) and adhering to the manufacturer’s guidelines. RNA integrity was verified by 1% agarose gel electrophoresis, while concentration and purity (A260/A280 ratio) were determined using a BioDrop™ spectrophotometer (Fisher Scientific, Needham, MA, USA).

Complementary DNA (cDNA) was generated from 2 µg of total RNA using the StaRT Reverse Transcription Kit (AnyGenes, Paris, France). Quantitative real-time PCR (qRT-PCR) was performed on a 7500 Fast Real-Time PCR System (Applied Biosystems, Waltham, MA, USA) employing TB Green^®^ Ex Taq™ (Tli RNase H Plus) reagents (Takara Bio Inc., Shiga, Japan) and 300 nM of specific prevalidated primers (AnyGenes, Paris, France). The amplification program consisted of an initial denaturation at 95 °C for 10 min, followed by 45 cycles of 95 °C for 10 s and 60 °C for 30 s, and a final melting curve analysis (95 °C for 10 s, 65 °C for 30 s, and 95 °C for 0 s), according to the manufacturer’s instructions. Expression of 18S rRNA served as an internal control. All reactions were performed in triplicate, and relative mRNA levels were quantified using the 2^−ΔΔCT^ method [19].

### 3.4. Protein Expression (tnf-α, il-1β, il-10, bad, bax, bak, bcl-2, mcl-1, inos, enos and nf-κb) and Variables Related to Apoptosis (Puma, Khloto)

Protein expression analysis was carried out by Western blot using specific primary antibodies against TNF-α (Endogen, Inc.—Woburn, MA, USA); IL-1β (PeproTech, Cranbury, NJ, USA); IL-6 (Cell Signaling Technology (CST), Danvers, MA, USA) , nitric oxide synthase isoforms I, II, and III (Chemicon International, Inc.—Merck KGaA, Darmstadt, Germany); NF-κB (Endogen, Inc. — Woburn, MA, USA); and IκB (Endogen, Inc.—Woburn, MA, USA).

Liver tissue samples were homogenized in a modified RIPA lysis buffer composed of PBS, Igepal, sodium deoxycholate (D5670-5G), 10% SDS, PMSF, 0.5 M EDTA, and 100 mM EGTA, supplemented with a protease inhibitor cocktail (Sigma #P-2714), PMSF (1 mM, #P7626), sodium orthovanadate (2 mM, #S6506), and sodium pyrophosphate (20 mM, #S6422). The homogenates were then sonicated and boiled for 10 min at 100 °C in a 1:1 ratio with loading buffer (100 mmol/L Tris-HCl, pH 6.8; 4% SDS; 20% glycerol; 0.1% bromophenol blue; and 200 mmol/L dithiothreitol).

Equal quantities of total protein (25 µg per sample) were separated on 10% Mini-PROTEAN^®^ TGX™ precast acrylamide gels (Bio-Rad Laboratories, Hercules, CA, USA) and subsequently transferred to PVDF membranes using a Trans-Blot^®^ Turbo™ Transfer System (Bio-Rad Laboratories, Hercules, CA, USA).

Membranes were immediately incubated in a blocking solution consisting of 5% non-fat dry milk prepared in 20 mM Tris (pH 7.5), 150 mM NaCl, and 0.01% Tween-20 for 1 h at 37 °C. Thereafter, they were incubated overnight (12 h) at 4 °C with rabbit polyclonal primary antibodies diluted 1:1000, followed by a 1 h incubation with a goat anti-rabbit IgG secondary antibody (Santa Cruz Biotechnology, Santa Cruz, CA, USA) at a 1:7000 dilution.

Immunoreactive bands were visualized by chemiluminescence using the Clarity Western ECL Substrate Kit (Bio-Rad Laboratories, Hercules, CA, USA) and ECL Plus reagents (Amersham Life Science Inc., Buckinghamshire, UK) on a Bio-Rad^®^ ChemiDoc MP Imaging System. Pre-stained molecular weight markers were employed for band size determination. GAPDH (1:5000; Santa Cruz Biotechnology, Santa Cruz, CA, USA) served as the loading control, and densitometric quantification of protein bands was performed with Bio-Rad^®^ Image Lab software Image Lab 6.0 (Bio-Rad Laboratories, Hercules, CA, USA).

### 3.5. Statistical Analysis

The experiment data was collected in an Access database designed for the project. To determine if the data followed a normal distribution, the Shapiro–Wilk test was used. To determine whether the results of the different experimental groups were homogeneous at baseline, the ANOVA-F test was performed. To determine whether the differences between the means of the different groups were statistically significant, a repeated measures ANOVA was performed. After rejecting the Null Hypothesis of equality of means by means of ANOVA, the Tukey test was used to compare means between different experiments. All data are expressed as mean ± the standard deviation of the mean. The analysis of results was carried out with the statistical package SPSS 23.

## 4. Results

### 4.1. General, Hemodynamic, and Gasometric Parameters

Our results showed high stability in gasometric and hemodynamic parameters between both, CONTROL and LIDO groups. No significant differences were found between groups regarding animal weight, anesthesia duration, or surgical time.

### 4.2. Liver Biopsy

We perform all our measurements in the liver biopsy we take in each porcine model at the 24 h time point.

### 4.3. Inflammatory Markers Analyzed by PCR in Liver

LRS with OLV increased hepatic expression of proinflammatory mediators.

TNF-α expression was significantly higher in the control group (CON) compared with the lidocaine (LIDO) and sham (SHAM) groups (*p* < 0.001). The difference between LIDO and SHAM was not significant (*p* = 0.526).

IL-1 levels were also elevated in the CON group compared with LIDO (*p* = 0.008) and SHAM (*p* = 0.004). A smaller but significant increase was observed in the LIDO group compared with SHAM (*p* = 0.003). MCP-1 values were significantly lower in the LIDO group than in CON (*p* = 0.004) and SHAM (*p* = 0.003) groups, while MCP-1 levels in CON were slightly higher than in SHAM (*p* = 0.078).

IL-10 values showed no significant differences among groups (*p* = 0.748).

**Figure 2:**
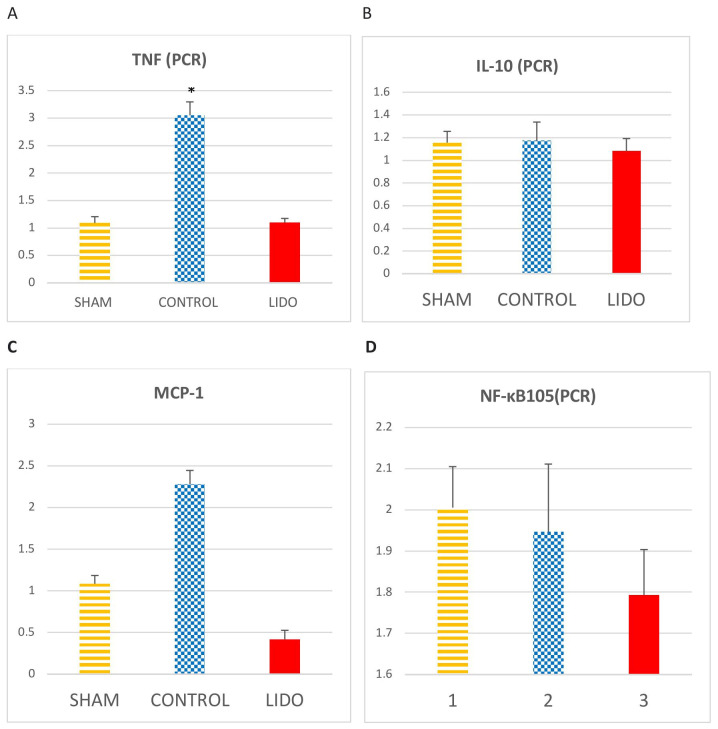
Hepatic inflammatory markers measured by PCR. Bar graphs show cytokine expression in liver biopsies obtained 24 h after surgery. Comparisons are shown between the control (CON), lidocaine (LIDO), and sham (SHAM) groups. (**A**) TNF-α; (**B**) IL-10; (**C**) MCP-1; (**D**) NF-κB. * *p* < 0.005 CON vs. SHAM. Abbreviations: TNF = tumor necrosis factor; IL = interleukin; MCP-1 = monocyte chemoattractant protein-1; NF-κB = nuclear factor kappa B.

NF-κB expression tended to decrease in the LIDO group compared with CON and SHAM, though not significantly (*p* = 0.749) (Table 1) (Figure 2).

**Table 1:** Hepatic inflammatory cytokines (PCR, 24 h post-surgery).

Marker	CON vs. SHAM	LIDO vs. CON	*p*-Value	Effect of Lidocaine
TNF-α	↑	↓	<0.001	Reduced TNF-α expression
IL-1	↑	↓	0.008	Reduced IL-1 expression
MCP-1	↑	↓	0.004	Decreased MCP-1 levels
IL-10	ns	ns	0.748	No significant difference
NF-κB	↑	↓	0.749	Trend toward lower activation

↑ increase; ↓ decrease; ns = not significant.

### 4.4. Inflammatory Markers Analyzed by WB Technique in Liver

Western blot analysis confirmed the PCR findings. TNF-α and IL-1 protein levels were increased in the CON group compared with SHAM and LIDO.

The anti-inflammatory cytokine IL-10 showed higher levels in the LIDO group compared with CON and SHAM, although the difference was not statistically significant (*p* = 0.055).

**Figure 3:**
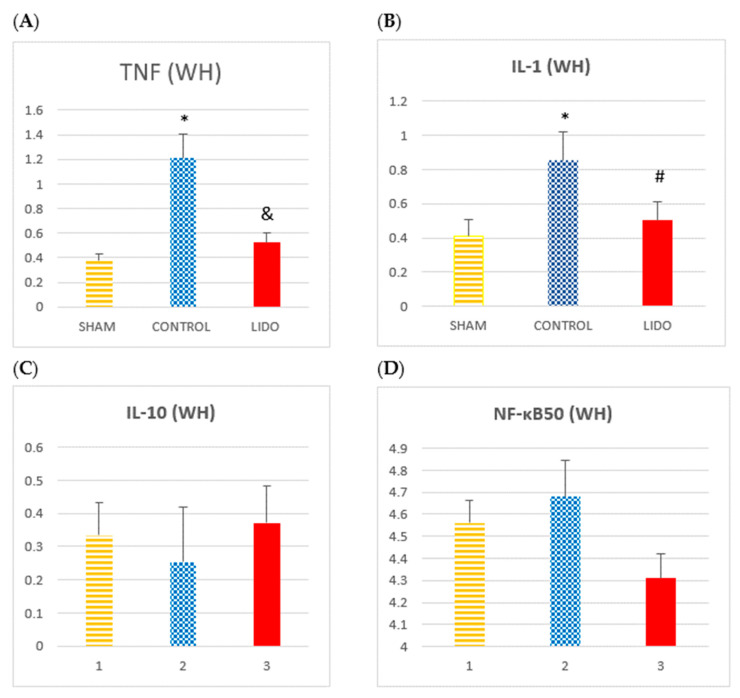

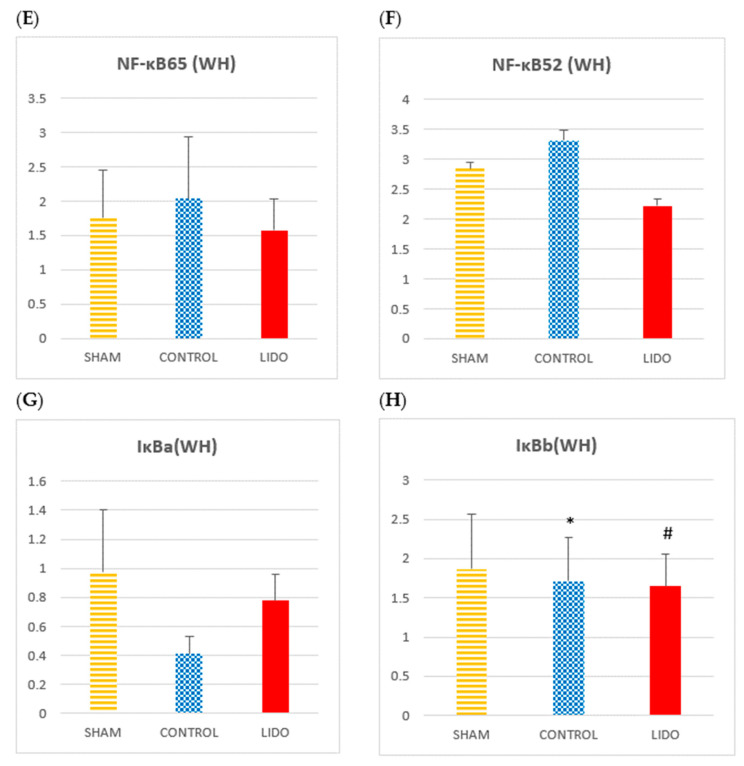
Hepatic inflammatory markers measured by Western blot. Bar graphs show protein expression of inflammatory mediators in liver biopsies collected 24 h after surgery. Comparisons are shown between the control (CON), lidocaine (LIDO), and sham (SHAM) groups. (**A**) TNF-α; (**B**) IL-1; (**C**) IL-10; (**D**) NF-κB50; (**E**) NF-κB65; (**F**) NF-κB52; (**G**) IκBα; (**H**) IκBβ. * *p* < 0.005 CON vs. SHAM; # *p* < 0.005 LIDO vs. SHAM; and & *p* < 0.005 LIDO vs. CON. Abbreviations: TNF = tumor necrosis factor; IL = interleukin; NF-κB = nuclear factor kappa B; IκB = inhibitor of NF-κB.

NF-κB subunits were elevated in the CON group compared with SHAM and LIDO. Both OLV groups (CON and LIDO) presented lower IκBβ expression than SHAM, and IκBβ levels were significantly lower in CON compared with LIDO (*p* = 0.006) (Table 2) (Figure 3).

**Table 2:** Protein expression of hepatic inflammatory markers (Western blot, 24 h).

Protein	CON vs. SHAM	LIDO vs. CON	*p*-Value	Effect of Lidocaine
TNF-α	↑	↓	<0.005	Reduced TNF-α
IL-1	↑	↓	<0.005	Lower IL-1 levels
IL-10	↓	↑	0.055	Trend to higher IL-10
NF-κB (p105/p65/p50)	↑	↓	0.049	Reduced NF-κB activation
IκBβ	↓	↑	0.006	Preserved inhibitor expression

↑ increase; ↓ decrease.

### 4.5. Nitric Oxide Metabolism

Inducible nitric oxide synthase (iNOS) was markedly increased in both CON and LIDO groups compared with SHAM (*p* = 0.01 and *p* = 0.005, respectively). However, iNOS levels were significantly higher in CON than in LIDO (*p* = 0.004).

**Figure 4:**
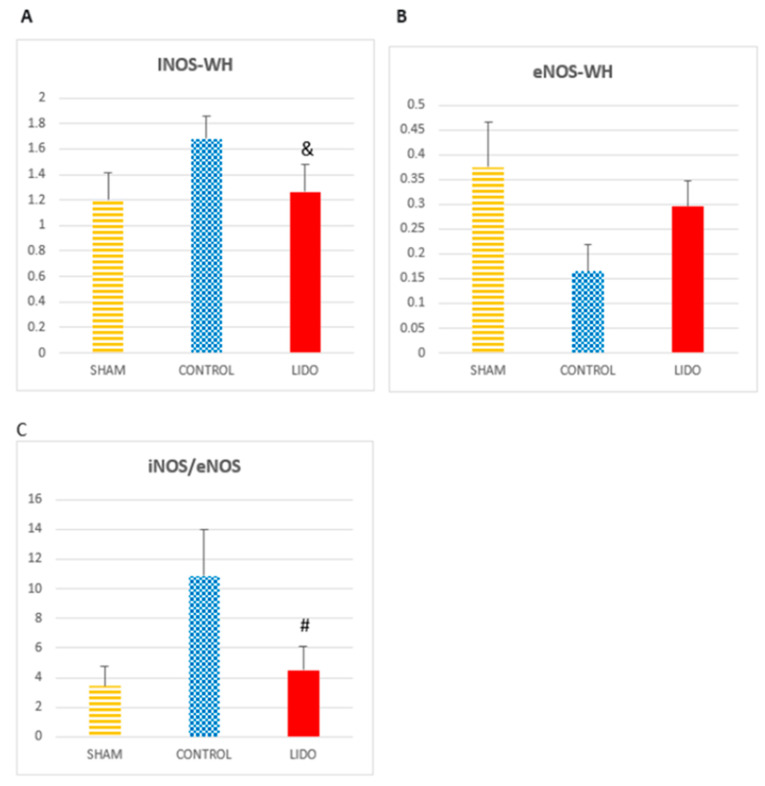
Nitric oxide (NO) metabolism in liver biopsies. Bar graphs show protein expression of nitric oxide-related enzymes in liver tissue 24 h after surgery. Comparisons are shown between the control (CON), lidocaine (LIDO), and sham (SHAM) groups. (**A**) eNOS expression; (**B**) iNOS expression; (**C**) iNOS/eNOS ratio. # *p* < 0.003 LIDO vs. SHAM; and & *p* < 0.003 LIDO vs. CON. Abbreviations: iNOS = inducible nitric oxide synthase; eNOS = endothelial nitric oxide synthase.

Endothelial nitric oxide synthase (eNOS) expression was decreased in CON and LIDO compared with SHAM (*p* = 0.006 and *p* = 0.001, respectively), with lower values in CON than LIDO (*p* = 0.011). Consequently, the iNOS/eNOS ratio was significantly higher in CON compared with both SHAM (*p* = 0.006) and LIDO (*p* = 0.005), indicating a more favorable nitric oxide balance with lidocaine treatment (Table 3) (Figure 4).

**Table 3:** Nitric oxide metabolism in liver biopsies (24 h).

Marker	CON vs. SHAM	LIDO vs. CON	*p*-Value	Effect of Lidocaine
iNOS	↑	↓	0.004	Reduced iNOS expression
eNOS	↓	↑	0.011	Preserved eNOS expression
iNOS/eNOS ratio	↑	↓	0.005	Improved NO balance

↑ increase; ↓ decrease.

### 4.6. Apoptosis

Markers of apoptosis showed increased expression of pro-apoptotic proteins in the control group. BAD and BAK values were significantly higher in CON compared with SHAM and LIDO (*p* = 0.004 and *p* = 0.004, respectively). In the LIDO group, these proteins were markedly reduced compared with CON (BAD: *p* = 0.004; BAK: *p* = 0.007). BAX and BCL-XL decreased in LIDO compared with the other groups, though not significantly. MCL-1 levels were lower in LIDO, but the difference was not significant (*p* = 0.631).

**Figure 5:**
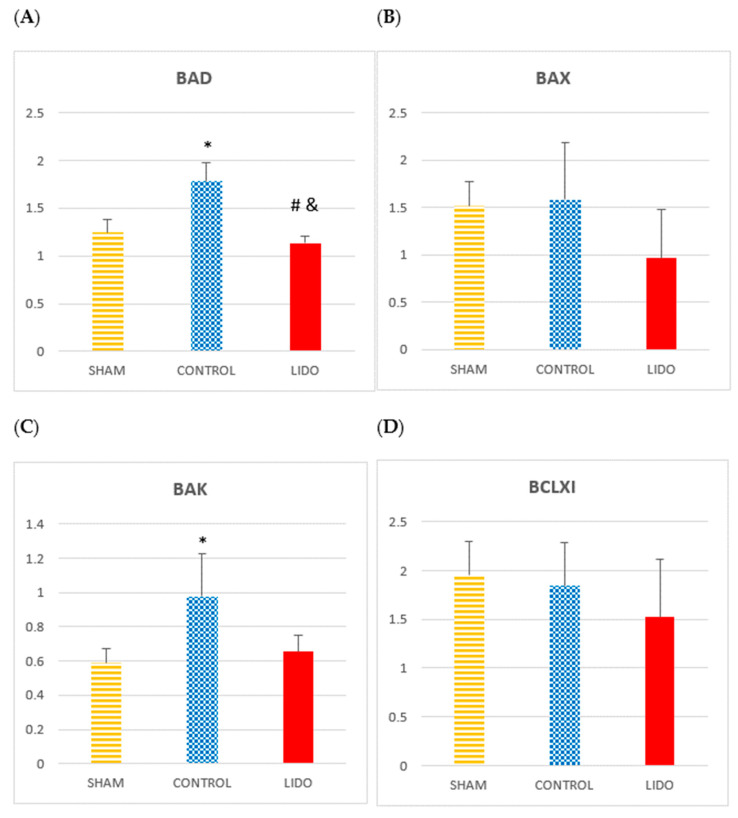

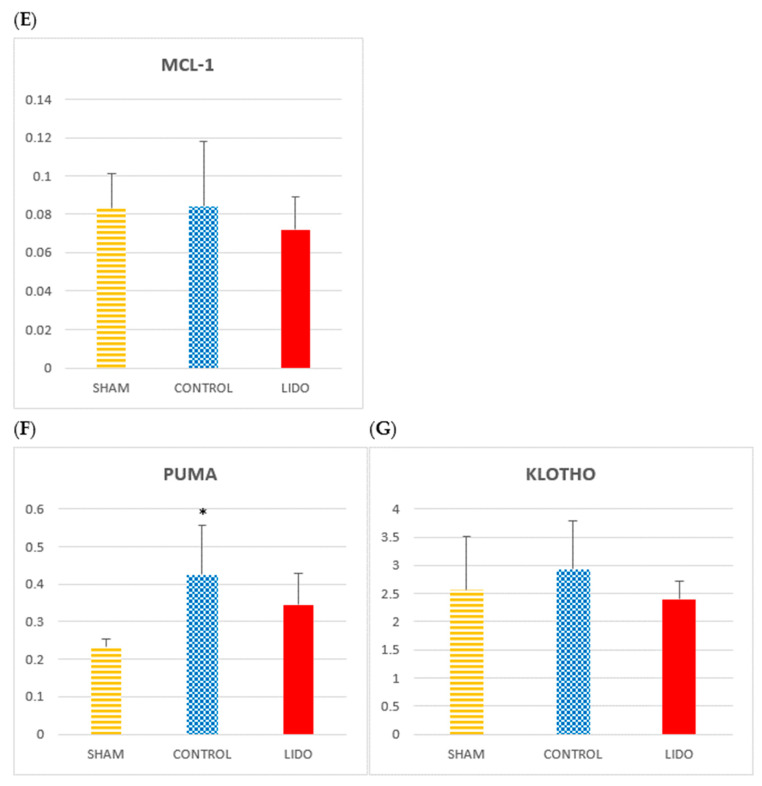
Apoptosis-related proteins in liver biopsies. Bar graphs show the expression of apoptotic mediators in liver samples obtained 24 h after surgery. Comparisons are shown between the control (CON), lidocaine (LIDO), and sham (SHAM) groups. (**A**) BAD; (**B**) BAX; (**C**) BAK; (**D**) BCL-XL; (**E**) MCL-1; (**F**) PUMA; (**G**) KLOTHO. * *p* < 0.003 CON vs. SHAM; # *p* < 0.003 LIDO vs. SHAM; and & *p* < 0.003 LIDO vs. CON. Abbreviations: BAD = Bcl-2-associated death promoter; BAX = Bcl-2-associated X protein; BAK = Bcl-2 homologous antagonist/killer; BCL-XL = Bcl-2-like protein X long isoform; MCL-1 = myeloid cell leukemia 1; PUMA = p53 upregulated modulator of apoptosis; KLOTHO = anti-aging protein with cytoprotective and antioxidant functions.

PUMA levels were significantly increased in CON and LIDO compared with SHAM (*p* = 0.004 and *p* = 0.007, respectively), with no difference between the two experimental groups. KLOTHO levels were highest in CON and lowest in LIDO, though the difference was not statistically significant (*p* = 0.423) (Table 4) (Figure 5).

**Table 4:** Apoptosis-related proteins in liver biopsies (24 h).

Marker	CON vs. SHAM	LIDO vs. CON	*p*-Value	Effect of Lidocaine
BAD	↑	↓	0.004	Reduced BAD expression
BAK	↑	↓	0.007	Reduced BAK expression
BAX	↑	↓ (ns)	0.631	Non-significant decrease
MCL-1	↓	↓ (ns)	0.631	No significant difference
PUMA	↑	↔	0.004	No difference vs. CON
KLOTHO	↑	↓	0.423	Non-significant decrease

↑ increase; ↓ decrease; ↔ no change; ns = not significant.

## 5. Discussion

Surgery increases proinflammatory cytokines, which leads to a systemic inflammatory response. Our group previously found that lidocaine could prevent the inflammatory damage caused by OLV and attenuate apoptosis [16]. In this study, we verified that the administration of intravenous lidocaine during LRS in a porcine experimental model is associated with an attenuation of the hepatic inflammatory response.

Inflammation is part of the pathogenesis of liver diseases and is regulated by inflammatory mediators [20]. Kupffer cells, hepatic macrophages, play a determining role in cellular stress and hepatic inflammation; they are considered key cells in the inflammatory process [21].

The liver plays a determining role in regulating the systemic inflammatory response, being especially sensitive to inflammatory changes [6,8]. Hepatocytes release inflammatory mediators after contact with perioperative cytokines, amplifying the perioperative inflammatory response [22].

In our study, we observed the relevance of the biotrauma associated with OLV; animals in the SHAM group (without OLV) developed a lower hepatic inflammatory response. This can be due to mechanical ventilation of the dependent lung and due to the IR process or surgical manipulation in the non-ventilated lung [23].

Multiple investigations have shown that lidocaine can modulate the perioperative systemic inflammatory response [14,15,24]; however, its protective effects on perioperative liver inflammation have not been so well studied.

### 5.1. Inflammation

Intravenous lidocaine reduces the adhesion of PMN to the endothelium and its migration to the site of inflammation, attenuates vascular inflammation, increases anti-inflammatory cytokines, and decreases inflammatory cytokines [25].

We observed an increase in the production of oxygen radicals and adhesion molecules, and we related it to an increase in interactions between PMN neutrophils and liver vascular endothelial cells that was attenuated by lidocaine administration. The role of MCP-1 as a regulator of the activation, migration, and infiltration of monocytes and macrophages to the site of inflammation is essential to start the inflammatory response [26]. In our study, the expression of MCP was attenuated in the LIDO group, and we believe this is one of the mechanisms by which lidocaine attenuated liver inflammation. Lin et al. obtained similar results in the prevention of hepatorenal damage in a septic rat model and found that lidocaine administration improved survival, decreased cell adhesion molecules, and MCP-1 [26]. We hypothesize that the action model of lidocaine in lung macrophage PMNs could be extrapolated to liver PMNs, so that its modulatory effect on inflammation could be directly related to the effects that lidocaine has on liver Kupffer cells. Reinforcing this hypothesis, in another experimental study, it was observed that lidocaine decreased the NF-kB signaling pathway in murine macrophages with lipopolysaccharide-induced sepsis [27]. NF-kB activation raised the expression of proinflammatory cytokines [28,29].

Perioperative liver inflammation increases NF-kB and TNFα liver expression [29]; there is research supporting that lidocaine blockade of the NF-kB pathway is dose-dependent [24,30].

Although in our study we did not show that the administration of lidocaine directly alters this signaling pathway, probably because such high doses were not reach as in the studies previously mentioned, we do appreciate that the cytoplasmic inhibitor of NF-kB (IKB), which blocks the activation and translocation to the nucleus of NF-kB, was decreased in the control group and did not change in the lidocaine group, so we think that part of the protective effect of lidocaine is due to the maintenance of levels of inhibitors of this pathway, this inhibition is probably related to the effect of NO on this IKB, as has been shown in other previous studies [28,31,32].

### 5.2. NO

The relationship between the increase in NO concentration in infection, inflammation, or sepsis is well known [33]. After liver injury, hepatocytes express inducible nitric oxide synthase (iNOS) with a consequent increase in NO production, which contributes to hepatocyte injury. iNOS has a proinflammatory action, decreases available NO, and causes oxidative stress [34,35]. Using a murine model of inflammation-related liver damage, Zhang et al. found that the increase in liver NO is attributable to a decrease in eNOS and an increase in iNOS, since iNOS knockout mice were protected against such liver injury [29].

Several experimental studies using macrophage cells [26,31,36] have shown how lidocaine inhibits NO production in a dose-dependent manner, apparently by suppressing L-arginine uptake. Furthermore, it inhibits iNOS, possibly involving voltage-gated sodium channels [26,36].

In other cell cultures [33], an inhibitory effect of TNF-alpha induced by eNOS activation has also been observed in lung microvascular endothelial cells, which prevents NO production and further propagation of inflammatory signaling [27]. However, at the hepatic level, there is no extensive bibliography in this regard.

In our work, the administration of lidocaine protected against the mismatch between iNOS/eNOS, mainly at the expense of the increase in eNOS that balances the decrease in iNOS in the LIDO group compared to the other groups. There is probably a compensation between both isoforms, and that is why NOx metabolism remains stable, demonstrating the hepatic protective effect of lidocaine on NO metabolism. Previously, our group showed that lidocaine provided a balance between both isoforms in both lungs [26]. There are previous works in which macrophages were trated with LPS to allow assessment of the significant inhibitory effects of lidocaine on iNOS transcription, making evident the anti-inflammatory capacity of lidocaine [26]. We believe that the fact that lidocaine suppresses the release of NO by activated macrophages could be the key to attenuating liver damage in the context of postoperative inflammatory liver damage.

Perhaps another protective effect of lidocaine observed in our study comes from the recognized protection that lidocaine has in IR phenomena, through decreased adhesion and migration of PMNs or inhibition of activated neutrophils [16,19].

### 5.3. Apoptosis

Apoptosis plays an important role in hepatocyte damage. Mechanisms of hepatic apoptosis are complicated by multiple signaling pathways [32]. In our study, we verified how the balance between pro- and anti-apoptotic mediators in liver biopsies from animals that had received lidocaine leaned towards anti-apoptosis. Lidocaine has been shown in the majority of the studies to have an antiapoptotic protective role in different inflammatory states [25,32,37,38]. The specific mechanisms by which lidocaine attenuates apoptosis are not clear; it is known that it blocks the extrinsic pathway of apoptosis by decreasing proinflammatory cytokines, especially TNFα, and blocking the intrinsic pathway through the decrease in the bcl-2 and PUMA family proteins. We believe that the decrease in PUMA expression was the major determinant of the antiapoptotic effect observed in our study. PUMA acts as a key mediator of the function of p53 in the cytosol, which is an important transcription factor that activates and regulates apoptosis. Other authors suggest that during early liver regeneration, PUMA downregulation may contribute to the suppression of apoptosis and inflammation [39]. They analyzed how after liver resection surgery, there is a progressive increase in the levels of PUMA, BAX and BCL-XL, which is related to an increase in apoptosis, in Kupffer cells induction and in neutrophils infiltration, as well as a higher number of fulminant hepatitis and mortality.

Our study reinforces the concept that perioperative modulation of inflammation with intravenous lidocaine could be integrated into personalized anesthesia protocols. By identifying patients with a higher risk of systemic inflammatory or hepatic injury—based on biomarkers such as NFkB activation or MCP-1 levels—clinicians may tailor lidocaine use as a precision medicine strategy.

## 6. Limitations

Our study is subject to certain limitations. First, inflammatory markers were assessed only 24 h after surgery. Additional time points might have provided a more detailed temporal profile of the hepatic response and the dynamics of lidocaine’s protective effect. Future studies that include more time-based measurements, for example, at 6, 48, and 72 h postoperatively, would be interesting and justified. Second, the lidocaine doses used were within clinically accepted ranges, which may explain differences compared with in vitro studies using higher concentrations. Finally, our experimental model involved healthy pigs; therefore, the results may not fully reflect the response in patients with pre-existing liver disease, who could exhibit different inflammatory and metabolic reactions. Future disease-model research will enhance clinical relevance.

## 7. Conclusions

Our results suggest that intraoperative use of intravenous lidocaine perfusion modulates the postoperative liver inflammatory response associated with the surgery, especially for the anti-inflammatory and antiapoptotic effects and for the protection of nitric oxide metabolism.

The results reinforced the potential role of intravenous lidocaine as part of a personalized medicine approach in thoracic surgery and future clinical trials to confirm lidocaine’s protective effects and to explore its role as a perioperative anti-inflammatory strategy in thoracic surgery.

## Data Availability

The original contributions presented in this study are included in the article. Further inquiries can be directed to the corresponding author.
